# U-Shaped Association of the Heart Rate Variability Triangular Index and Mortality in Hemodialysis Patients With Atrial Fibrillation

**DOI:** 10.3389/fcvm.2021.751052

**Published:** 2021-11-29

**Authors:** Matthias C. Braunisch, Christopher C. Mayer, Stanislas Werfel, Axel Bauer, Bernhard Haller, Georg Lorenz, Roman Günthner, Julia Matschkal, Quirin Bachmann, Stephan Thunich, Michaela Schlegl, Maximilian Ludwig, Christopher Holzmann-Littig, Tarek Assali, Martin Pachmann, Claudius Küchle, Lutz Renders, Siegfried Wassertheurer, Alexander Müller, Georg Schmidt, Uwe Heemann, Marek Malik, Christoph Schmaderer

**Affiliations:** ^1^Department of Nephrology, School of Medicine, Klinikum Rechts der Isar, Technical University of Munich, Munich, Germany; ^2^Center for Health and Bioresources, Biomedical Systems, AIT Austrian Institute of Technology GmbH, Vienna, Austria; ^3^University Hospital for Internal Medicine III, Medical University Innsbruck, Innsbruck, Austria; ^4^Department of Cardiology, Munich University Clinic, DZHK (German Centre for Cardiovascular Research), Ludwig-Maximilians University, Munich, Germany; ^5^School of Medicine, Klinikum Rechts der Isar, Institute of Medical Informatics, Statistics and Epidemiology (IMedIS), Technical University of Munich, Munich, Germany; ^6^School of Medicine, Deutsches Herzzentrum München, Technical University of Munich, Munich, Germany; ^7^Nephrocare DIZ-München, Rindermarkt, Munich, Germany; ^8^School of Medicine, Klinik für Innere Medizin I, Klinikum Rechts der Isar, Technical University of Munich, Munich, Germany; ^9^National Heart and Lung Institute, Imperial College London, London, United Kingdom; ^10^Department of Internal Medicine and Cardiology, Faculty of Medicine, Masaryk University, Brno, Czechia

**Keywords:** atrial fibrillation, heart rate variability triangular index, HRVi, cardiovascular mortality, hemodialysis, risk prediction

## Abstract

**Background:** Atrial fibrillation (AF) is common in hemodialysis patients and contributes to increased mortality. We aimed to examine heart rate variability triangular index (HRVI) in hemodialysis patients with AF as it has recently been reported to predict mortality in AF patients without kidney disease.

**Methods:** A total of 88 patients on hemodialysis with a medical history of AF or newly diagnosed AF underwent 24-h electrocardiography recordings. The primary endpoint of cardiovascular mortality was recorded during a median follow up of 3.0 years. Risk prediction was assessed by Cox regression, both unadjusted and adjusted for the Charlson Comorbidity Index and the Cardiovascular Mortality Risk Score.

**Results:** Median age was 76 years, median dialysis vintage was 27 months. Altogether, 22 and 44 patients died due to cardiovascular and non-cardiovascular causes. In 55% of patients AF was present during the recording. Kaplan-Meier plots of HRVI quartiles suggested a non-linear association between HRVI, cardiovascular, and all-cause mortality which was confirmed in non-linear Cox regression analysis. Adjusted linear Cox regression revealed a hazard ratio of 6.2 (95% CI: 2.1–17.7, *p* = 0.001) and 2.2 (95% CI: 1.3–3.8, *p* = 0.002) for the outer quartiles (combined first and fourth quartile) for cardiovascular and all-cause mortality, respectively. Patients in the first quartile were more likely to have sinus rhythm whereas patients in the fourth quartile were more likely to have AF.

**Conclusions:** We found a U-shaped association between HRVI and mortality in hemodialysis AF patients. The results might contribute to risk stratification independent of known risk scores in hemodialysis AF patients.

## Introduction

End-stage kidney disease patients on hemodialysis have a markedly increased risk of cardiovascular morbidity and mortality (1). In long term 6-month recordings with implantable loop recorders, atrial fibrillation (AF) was detected in up to 41% of hemodialysis patients (2). Dialysis-specific factors such as a higher ultrafiltration is associated with a higher incidence of AF (3). The incidence and prevalence of AF are higher in hemodialysis patients than in the general population (4, 5). This is contributed by multiple factors including age, dialysis vintage, left atrial dilatation, and high overall disease burden of hemodialysis patients (4, 6). Among hemodialysis patients, those suffering from AF have higher morbidity and mortality (7). AF is therefore a common problem in hemodialysis patients and contributes to the increased cardiovascular risk (7).

Rapid shifts of electrolytes, plasma volume and acidosis changes impair electrophysiology and expose hemodialysis patients to an increased risk of arrhythmogenic conditions (8). Peri-dialytic fluid and electrolyte flux stimulates a sympathetic reaction responsible for the increased prevalence of AF during and immediately following hemodialysis (2).

Analysis of the cardiac autonomic nervous system by non-invasive assessment of the heart rate variability (HRV) has been restricted to patients without AF, thus, limiting risk prediction in AF patients. In patients without kidney disease, cardiac autonomic dysfunction was associated with a higher AF incidence during 19 years of follow-up (9). The atria have a close autonomic innervation and the AV node is susceptible to input form the autonomic nervous system (10–13). Therefore, the autonomic nervous system is likely also involved in AF. Overall, data on risk prediction in hemodialysis patients with AF is scarce and primarily focused on anticoagulation agents (14–16).

Recent data from the Swiss-AF study reported that in AF patients without kidney disease, heart rate variability triangular index (HRVI) predicted cardiovascular mortality (17) and identified clinically silent strokes (18). HRVI is an estimate of total variability of RR intervals and approximates sympathovagal imbalance.

We thus hypothesized that an association of HRVI with mortality might also be present in hemodialyzed AF patients.

## Materials and Methods

### Study Design

**Figure 1:**
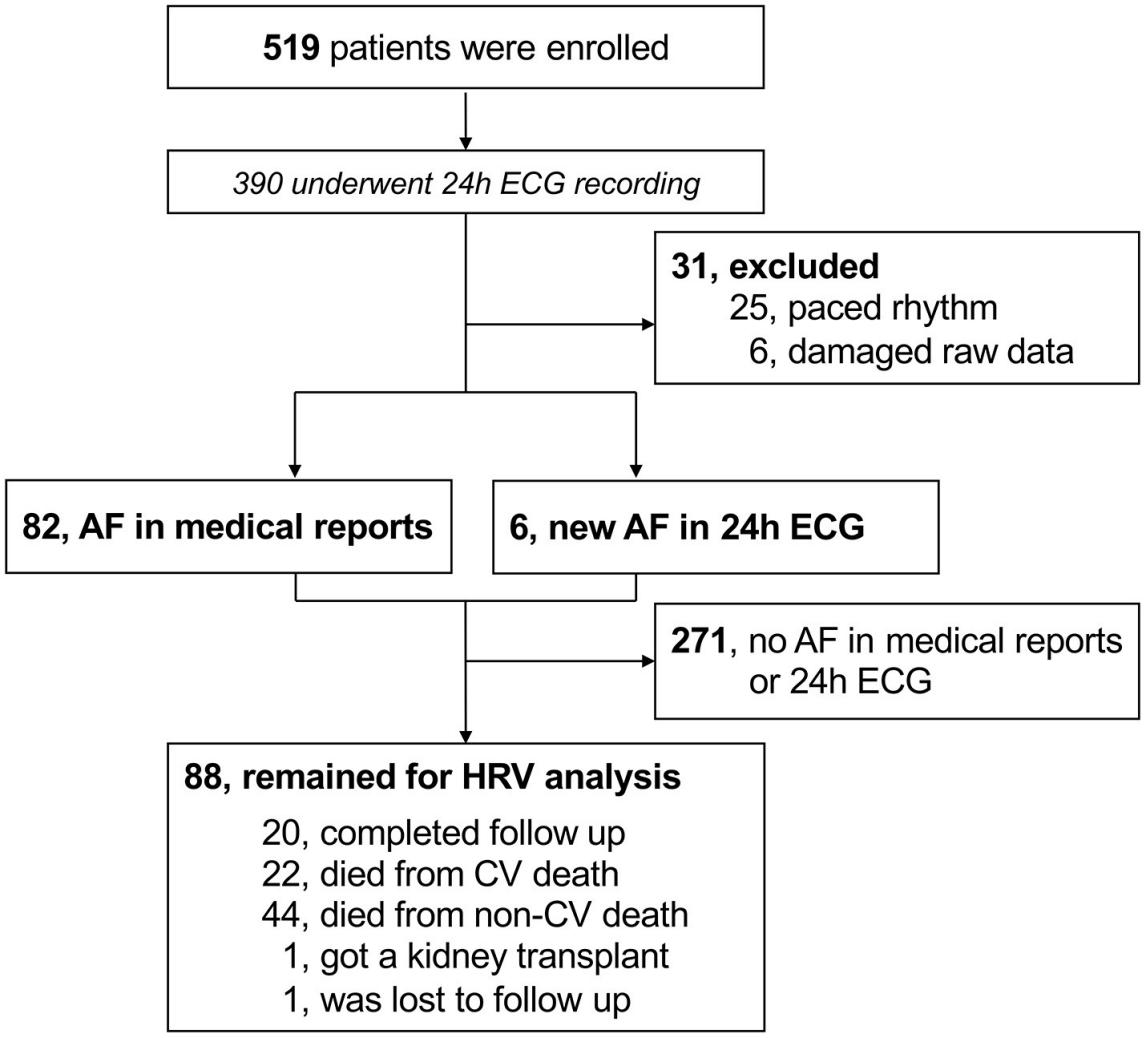
Flow-chart of participants. AF, atrial fibrillation; CV, cardiovascular; ECG, electrocardiogram; HRV, heart rate variability.

The study investigated the “rISk strAtification in end-stage Renal disease” (ISAR)-cohort, obtained in a multicenter, prospective longitudinal observational cohort study (ClinicalTrials.gov; identifier number: NCT01152892) (19). The study protocol, conforming to the ethical guidelines of the Helsinki Declaration, was approved by the Medical Ethics Committee of the Klinikum Rechts der Isar of the Technical University Munich and of the Bavarian State Board of Physicians and is compliant with the STROBE guidelines. Patients were recruited from 17 hemodialysis centers in the greater Munich area between April 2010 and January 2014. All participants gave informed written consent. Inclusion criteria were age ≥18 years and dialysis vintage ≥90 days (19). Patients were excluded if pregnant or if suffering from ongoing infection or malignancy with a life expectancy ≤ 24 months (19). Out of the 519 patients meeting inclusion criteria, 390 consented to undergo 24-h Holter electrocardiogram (ECG) recording. Out of these patients, 25 were excluded due to paced rhythm while 6 other patients had technically unsuitable ECG data. In 271 patients, AF was absent both in the medical reports and in the 24-h ECGs. The subsequent HRV analysis utilized the remaining 88 patients (corresponding flow-chart shown in Figure 1).

### Clinical Characteristics

Baseline demographic and clinical data were obtained from dialysis protocols and medical records. Medical records were screened for the diagnosis of AF and, where specified, coded into persistent/permanent or paroxysmal. Blood chemistry parameters were obtained prior to a midweek dialysis session. Comorbidities were assessed using an adapted version of the *Charlson Comorbidity Index* that has previously been validated for mortality prediction in hemodialysis patients (20). The index assigns numerical weights to comorbid conditions that range between 0 and 21 (20). Further, to assess cardiovascular mortality risk, the *Cardiovascular Mortality Risk Score* was calculated which has previously been developed and validated for the prediction of 2-year cardiovascular mortality in hemodialysis patients (21). The cardiovascular mortality risk score ranges between −11 and 39 points (21). The stroke risk factors are summarized in the CHA_2_DS_2_-VASc [Congestive heart failure, Hypertension, Age ≥75 years, Diabetes mellitus, Stroke, Vascular disease, Age 65–74 years, Sex category (female)] score (22).

### Endpoints

Mortality was assessed using medical records, databases of individual dialysis centers, or by contacting the attending physician and/or the next of kin. Using this information, the ISAR Endpoint Committee classified the underlying causes of death (19). For the purposes of the present study, cardiovascular and all-cause mortality was considered as the primary and secondary endpoint, respectively.

### Electrocardiography

In each patient, a 24-h 12-lead ECG recording was obtained using the Lifecard CF digital Holter recorder (Delmar Reynolds/Spacelabs Healthcare, Nuremberg, Germany). All recordings started before a mid-week dialysis session. Reference ECG annotations, RR-interval measurements, and artifact elimination were performed using the software tools of the equipment (Pathfinder, Delmar Reynolds/Spacelabs Healthcare, Nuremberg, Germany; V9.027) (23). Afterwards, all RR-intervals of normal heart beats were exported to Matlab R2020a (the MathWorks, Inc., Natick, MA) and HRV parameters were computed using the 24-h RR interval series according to established standards (24). HRVI was calculated by dividing the total number of normal-to-normal (NN) intervals (HRVI numerator) by the number of NN-intervals in the modal 1/128-s bin (HRVI denominator) according to guidelines (24). Additional HRV parameters included expressions of overall long-term variability by the standard deviation of all NN intervals (SDNN), the standard deviation of the averages of NN intervals in all 5 min segments (SDANN), and the ultra-low frequency power range of ≤ 0.003 Hz (ULF); and expressions of short-term variability by the square root of the mean square of differences between adjacent NN intervals (RMSSD). Initially, AF episodes were detected and quantified in the 24-h recordings by the AF algorithm integrated in the Pathfinder software. Additionally, all 24-h ECGs were reviewed for the presence of AF manually. Electrocardiographic diagnosis of AF was made in agreement with the definition of the European Society of Cardiology, i.e., irregularly irregular RR-intervals, absence of distinct repeating P waves, and irregular atrial activations (22). If AF was confirmed in the manual review the relative duration of AF during the recording indicated by the pathfinder software was recorded. Paroxysmal AF was defined as an AF burden ≤ 20% in the 24-h recording.

### Statistical Analysis

Categorical data are presented as frequencies and percentages. Continuous variables are expressed as mean ± standard deviation (SD) for normally distributed variables and as median and interquartile range (IQR) for variables with skewed distribution. To test for group differences, χ^2^ test was used for categorical variables and independent samples *t*-test or Mann-Whitney-*U* test for continuous variables, as appropriate.

In survival analysis, we first used Kaplan-Meier curves comparing quartiles to reveal possible violations of proportional hazard assumptions, thus identifying non-linear (non-proportional) associations. Subsequently, we fitted univariate and adjusted Cox regression models using penalized spline transformation with two degrees of freedom on HRVI (survival and splines packages). Adjusted models accounted for the *Charlson Comorbidity Index* and the *Cardiovascular Mortality Risk Score*. To overcome interpretation limitations of non-linear associations, values were dichotomized when in the first and forth quartile (outer quartiles) or in the second and third quartile (inner quartiles). Subsequently the dichotomized parameters were analyzed with regular univariate, and adjusted Cox regression models to estimate hazard ratios (HRs) for respective groups. Median follow-up was assessed by reverse Kaplan-Meier (25).

The predictive performance of the 24-h measurement vs. 5 min segments for HRVI and SDNN were compared using Harrell's C-index. For this purpose, 1000 adjusted Cox regressions for cardiovascular mortality with randomly selected 5 min intervals from the 24-h recordings were calculated. The mean ± SD C-indices were then compared with the C-index of the Cox regression model based on the 24-h measurement. Descriptive comparison of significant *p*-values for linear Cox regressions with 1000 randomly selected 5 min intervals were stratified for sinus rhythm and AF.

Kruskal-Wallis test was used to compare AF percentages amongst HRVI quartiles. *Post-hoc* analysis was performed with the Wilcoxon test and adjusted for multiple testing using the Bonferroni method.

Multivariable binary logistic regression with AF as dependent variable and the HRVI numerator and denominator as independent variables was used to obtain odds ratios with corresponding 95% confidence interval.

All tests were conducted two-sided and *p* < 0.05 were considered significant. Statistical analysis was performed using R version 4.0.2 (R Foundation for statistical Computing, Vienna, Austria).

## Results

### Patient Characteristics

The final study population included 88 patients (25 women) with a median follow-up of 3.0 years (IQR 1.5–5.8) (Figure 1). The median age was 76.4 years (70.6–80.6). The median dialysis vintage was 27.0 months (13.8–60.2). ß-blockers were taken by 64 patients (72.7%). Amiodarone was prescribed to 4 patients (4.5%) (Table 1). Sex differences are listed in Supplementary Table 1. Female patients had a significant higher dialysis vintage, systolic blood pressure, had significantly less frequently a history of myocardial infarction and coronary heart disease and were significantly less likely to smoke (Supplementary Table 1). Table 2 shows HRV measurements. Median HRVI was 26.3, quartiles of HRVI ranged from ≤ 18.245 (*n* = 22), 18.246–26.279 (*n* = 22), 26.280–34.307 (*n* = 22), and ≥34.08 (*n* = 22). Patients in the external quartiles were not significantly different from patients in the inner quartiles, except for heart rate which was significantly higher in patients in the inner quartiles and a history of myocardial infarction which was significantly more often present in patients in the outer quartiles (Table 1).

**Table 1:** Baseline characteristics.

	**HRVI quartiles**			
	**Total (*n* = 88)**	**Inner (*n* = 44)**	**Outer (*n* = 44)**	***P***
Age (years)	75.7 (±8.5)	75.1 (±9.4)	76.4 (±7.5)	0.48
Sex (female)	25 (28.4%)	10 (22.7%)	15 (31.8%)	0.34
Body mass index (kg/m^2^)	25.6 (23.0–28.4)	25.4 (23.3–28.9)	25.8 (23.0–27.9)	0.91
Dialysis vintage (months)	27.0 (13.8–60.2)	27.0 (12.5–58.2)	27.5 (14.8–62.8)	0.61
Ultrafiltration rate (mL/h)	518.7 (±273.3)	511.1 (±275.4)	526.4 (±274.2)	0.79
Net ultrafiltration (L)	1.7 (±1.3)	1.8 (±1.2)	1.6 (±1.3)	0.56
Heart rate (bpm)	73.0 (±13.5)	76.9 (±13.0)	69.0 (±12.8)	0.005
Systolic blood pressure (mmHg)	130.5 (±25.5)	125.9 (±27.9)	135.1 (±22.4)	0.092
Diastolic blood pressure (mmHg)	67.9 (±15.3)	67.6 (±14.8)	68.2 (±15.9)	0.86
Kt/V	1.34 (±0.32)	1.36 (±0.28)	1.33 (±0.36)	0.62
Blood urea nitrogen (mg/dL)	58.5 (±17.4)	58.1 (±18.4)	59.0 (±16.5)	0.83
Phosphate (mmol/L)	1.68 (1.39–2.01)	1.69 (1.40–2.10)	1.60 (1.35–1.99)	0.43
Total calcium (mmol/L)	2.31 (±0.17)	2.29 (±0.17)	2.33 (±0.17)	0.26
Calcium *x* phosphate (mmol^2^/L^2^)	4.06 (±1.67)	4.24 (±2.03)	3.88 (±1.20)	0.31
Creatinine (mg/dL)	7.1 (±2.3)	7.3 (±2.3)	6.9 (±2.3)	0.43
High-sensitivity CRP (mg/dL)	0.58 (0.22–1.20)	0.57 (0.22–1.30)	0.59 (0.26–0.98)	0.92
Albumin (g/dL)	3.90 (3.70–4.20)	3.90 (3.70–4.11)	3.90 (3.67–4.20)	0.99
Parathyroid hormone (pg/mL)	226.5 (128.5–359.6)	246.0 (131.9–359.0)	197.0 (126.0–334.3)	0.61
Leukocytes (G/L)	7.01 (±2.04)	6.70 (±1.87)	7.32 (±2.18)	0.16
Total cholesterol (mg/dL)	173.6 (±47.6)	172.3 (±36.2)	174.9 (±57.0)	0.80
Charlson comorbidity index (0–21)	6.0 (4.0–8.0)	6.0 (4.0–8.0)	6.0 (4.0–8.0)	0.68
Cardiovascular mortality risk score (−11 to 39)	14.0 (10.0–17.0)	14.0 (9.0–18.0)	14.0 (11.0–16.0)	0.75
Diabetes mellitus	38 (43.2%)	19 (43.2%)	19 (43.2%)	1.00
History of myocardial infarction	25 (28.4%)	7 (15.9%)	18 (40.9%)	0.017
Left ventricular hypertrophy	30 (34.1%)	13 (29.5%)	17 (38.6%)	0.50
Left ventricular ejection fraction (%), *n* = 22	47 (±16)	45 (±17)	50 (±15)	0.48
Heart failure	25 (28.4%)	11 (25.0%)	14 (31.8%)	0.64
Peripheral artery disease	26 (29.5%)	15 (34.0%)	11 (25.0%)	0.48
Hypertension	84 (95.5%)	43 (97.8%)	41 (93.2%)	0.62
Coronary heart disease	39 (44.3%)	16 (36.4%)	23 (59.0%)	0.20
Cerebrovascular disease	17 (19.3%)	9 (20.5%)	8 (18.2%)	0.78
Smoking (ever)	14 (15.9%)	10 (22.7%)	4 (9.0%)	0.14
CHA_2_DS_2_-VASc score	3.5 (3.0–4.0)	3.0 (3.0–5.0)	4.0 (3.0–5.0)	0.28
Anticoagulation for hemodialysis				0.71
Heparin	73 (83.0%)	37 (84.0%)	36 (81.8%)	
Low-molecular-weight heparin	12 (13.6%)	5 (11.4%)	7 (15.9%)	
Argatroban	3 (3.4%)	2 (4.5%)	1 (2.3%)	
ß-blocker	64 (72.7%)	31 (70.5%)	33 (75.0%)	0.81
Additional anticoagulation				0.49
None	54 (61.4%)	29 (65.9%)	25 (56.8%)	
Vitamin K antagonists	30 (34.1%)	14 (31.8%)	16 (36.4%)	
Low-molecular-weight heparin	4 (4.5%)	1 (2.3%)	3 (6.8%)	
Antiplatelet therapy				0.74
None	37 (42.0%)	21 (47.7%)	16 (36.4%)	
ASS	42 (47.7%)	19 (43.2%)	23 (52.3%)	
ADP receptor blocker	4 (4.5%)	2 (4.5%)	2 (4.5%)	
Dual antiplatelet therapy	5 (5.7%)	2 (4.5%)	3 (6.8%)	
Amiodarone	4 (4.5%)	1 (2.3%)	3 (6.8%)	0.62
Antihypertensive medication	83 (94.3%)	41 (93.2%)	42 (95.5%)	1.0

*Results are presented as mean (±SD) and median (interquartile range) for normally and non-normally distributed data, respectively; categorical data as total number (percentage). P-values present the results of group-wise comparisons of patients within the inner and outer quartiles of heart rate variability triangular index (HRVI). CHA_2_DS_2_-VASc, Congestive heart failure, Hypertension, Age ≥75 years, Diabetes mellitus, Stroke, Vascular disease, Age 65–74 years, Sex category (female)*.

**Table 2:** Measures of heart rate variability.

**Parameter**	**Unit**	**Value**
HRVI		28.3 ±12.7
SDNN	ms	102.0 (77.5–136.9)
SDANN	ms	83.1 ± 28.0
RMSSD	ms	33.5 (16.2–60.9)
ULF	ms^2^	3970.1 (2460.4–6020.8)

*Mean ± standard deviation or median and interquartile range, as appropriate. HRVI, HRV triangular index: number of NN intervals over the number of NN-intervals in the modal bin; SDNN, standard deviation of all NN intervals; SDANN, standard deviation of the averages of NN intervals in all 5-min segments of 24 h recording; RMSSD, square root of the mean square of differences between adjacent NN intervals; ULF, ultra-low frequency*.

In 6 patients (2.2%) AF was newly diagnosed using the 24-h ECG (Figure 1). At baseline, 42 (47.7%), 6 (6.8%), and 40 (45.5%) patients presented with permanent AF, paroxysmal AF, and sinus rhythm, respectively. The median percentage of AF burden in the 24-h recordings was 99.7% (87.3–99.9), 3.0% (1.1–3.8), and 0% (0–0) in patients with permanent AF, paroxysmal AF, and sinus rhythm, respectively. Of the 40 patients in sinus rhythm, paroxysmal and permanent AF had been previously documented in medical reports of 25 and 13 patients, respectively. In two patients, the type of the previously documented AF was not specified.

In comparison to the overall ISAR cohort, the patient population of the current study was significantly older, sicker and had higher cardiovascular mortality risk scores and shorter dialysis vintage (Supplementary Table 2).

### Anticoagulation

Overall, the median CHA_2_DS_2_-VASc score was 3.5 (3.0–4.0). A CHA_2_DS_2_-VASc score of ≥2 was present in 87 patients (98.9%). Anticoagulation for dialysis was prescribed in 73 (83.0%) with heparin, in 12 (13.6%) with low-molecular-weight heparin, and in 3 (3.4%) with argatroban. Additional anticoagulation was prescribed in 34 patients (38.6%), with 30 (34.1%) patients taking vitamin K antagonists and 4 (4.5%) taking low-molecular-weight heparin. No novel oral anticoagulants were prescribed. Antiplatelet therapy was present in 42 patients (47.7%) on acetylsalicylic acid, 4 (4.5%) on adenosine diphosphate (ADP) receptor inhibitors, and 5 (5.7%) on dual antiplatelet therapy.

### Association of HRVI and Mortality

Altogether, 22 and 44 patients died due to cardiovascular and non-cardiovascular causes, respectively. Details of cardiovascular and non-cardiovascular mortality are presented in Supplementary Table 3. One patient was censored due to kidney transplantation and one patient was lost to follow-up.

**Figure 2:**
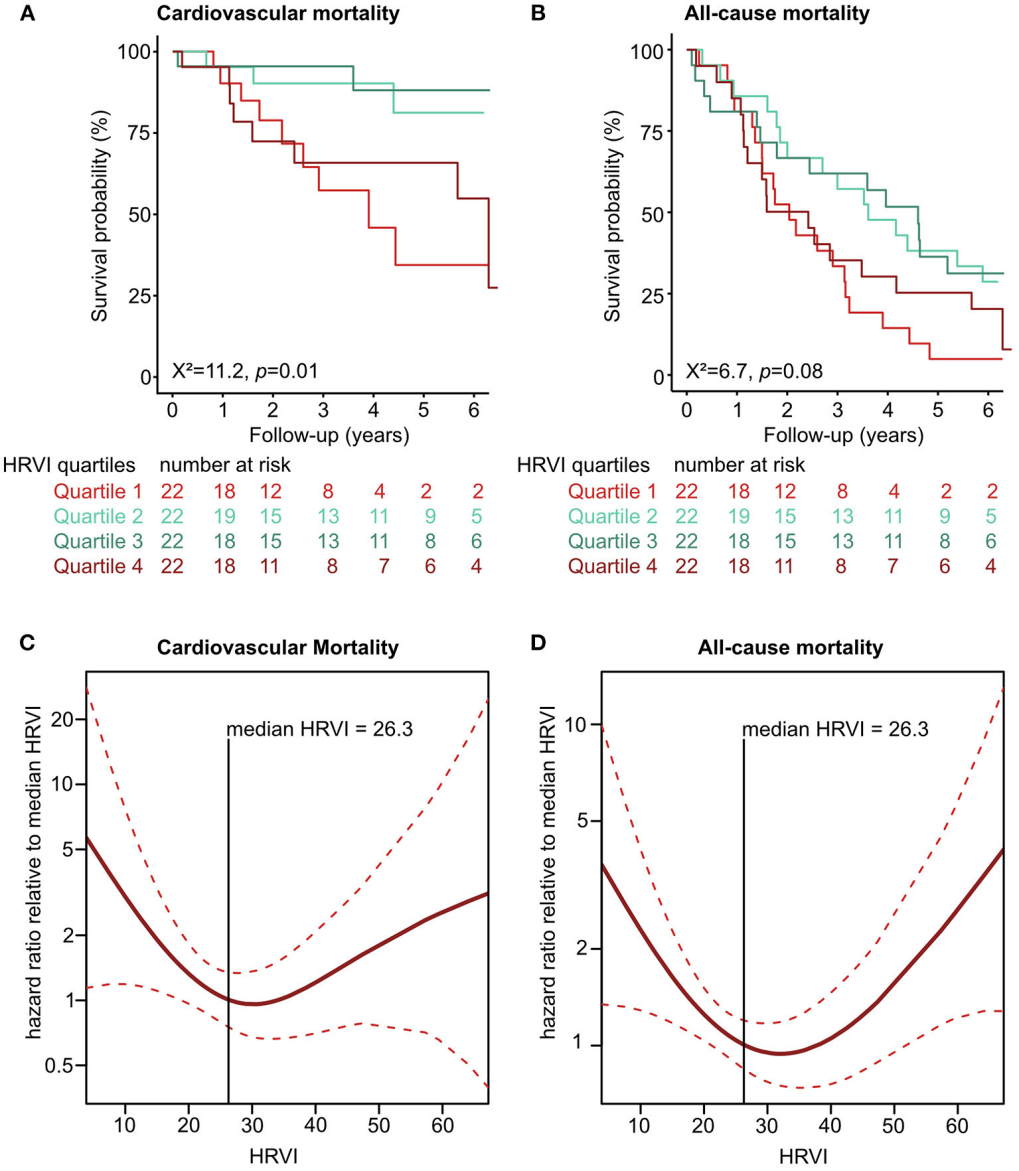
Non-linear association of HRVI with mortality. Univariate association of HRVI with cardiovascular **(A)** and all-cause mortality **(B)** in Kaplan-Meier analyses. Non-linear univariate Cox regression analysis using penalized smoothing splines for cardiovascular **(C)** and all-cause mortality **(D)**. HRVI, heart rate variability triangular index.

**Figure 3:**
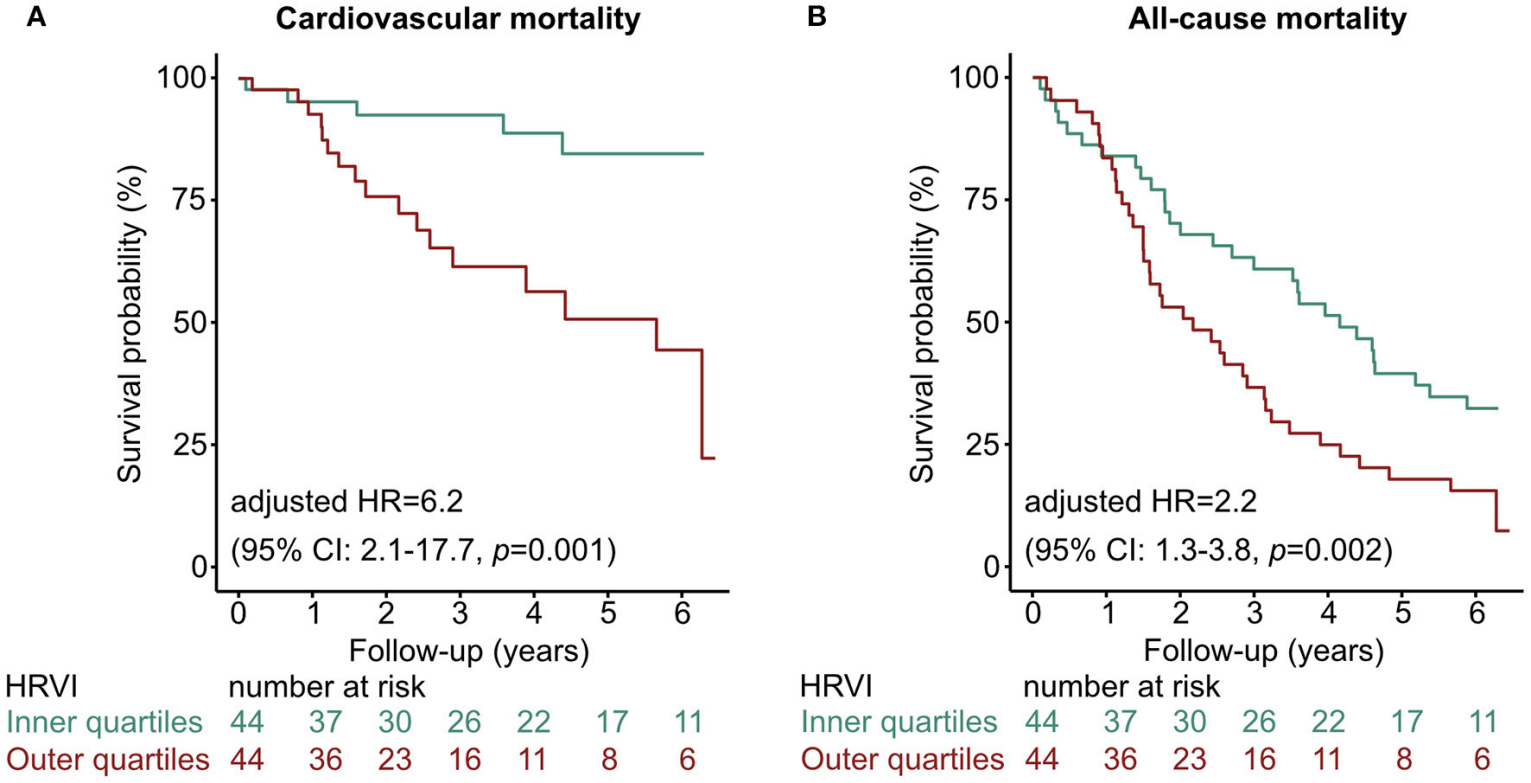
Kaplan-Meier curves of the inner vs. outer quartiles of HRVI for **(A)** cardiovascular mortality and **(B)** all-cause mortality. Hazard ratio after adjustment for the *Charlson Comorbidity Index* and the *Cardiovascular Mortality Risk Score*. CI, confidence interval; HR, hazard ratio; HRVI, heart rate variability triangular index.

**Figure 4:**
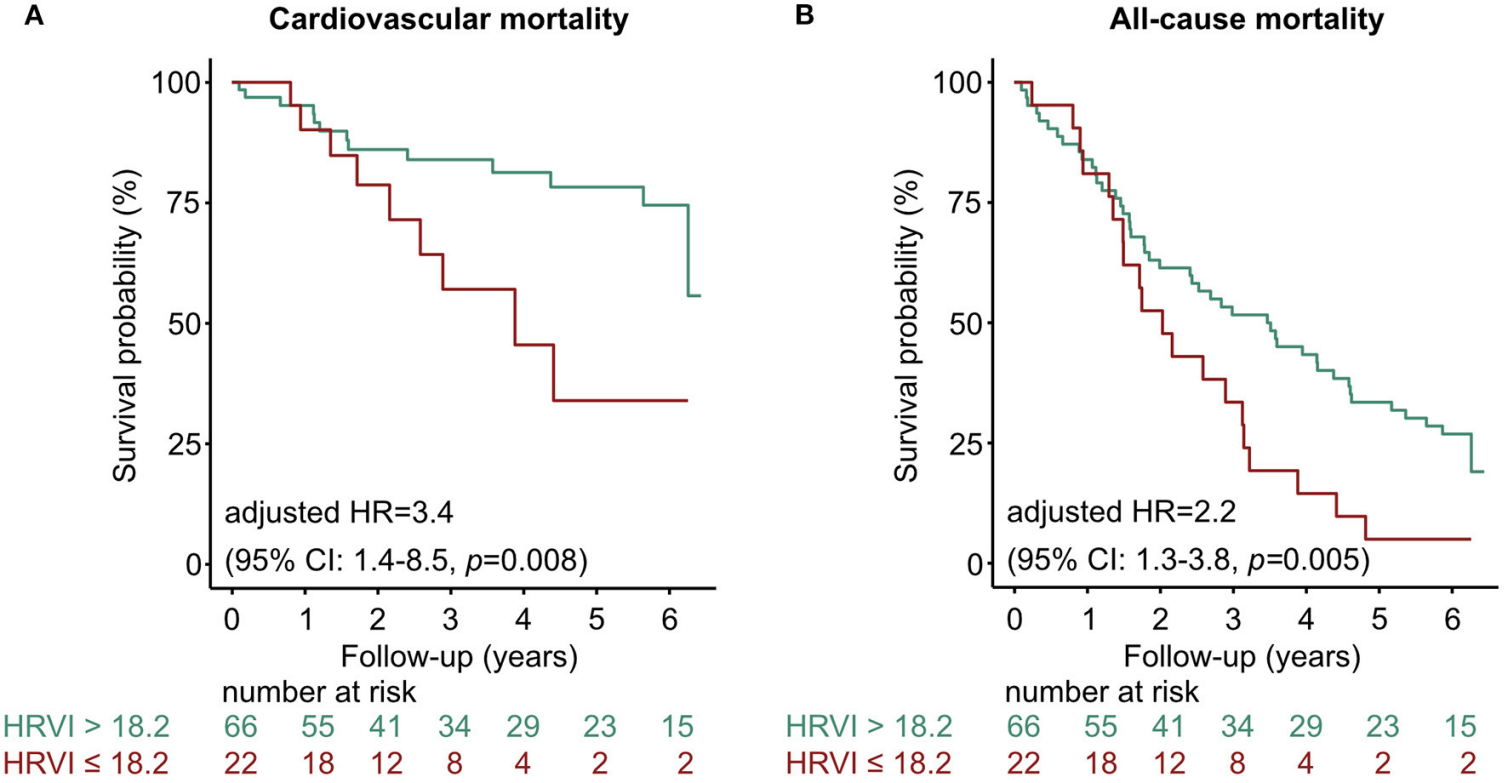
Kaplan-Meier curves stratified at the border of the first HRVI quartile for **(A)** cardiovascular and **(B)** all-cause mortality. The 3-year cardiovascular mortality rate was 31.8 and 13.6% if HRVI was ≤ 18.246 and >18.246, respectively. The 6-year cardiovascular mortality rate was 40.9 and 18.2% if HRVI was ≤ 18.246 and >18.246, respectively. For all-cause mortality the 3-year mortality rate was 63.4 and 45.5%, and the 6-year mortality rate was 90.9 and 68.2% if HRVI was ≤ 18.246 and >18.246, respectively. Hazard ratios are adjusted for the *Charlson Comorbidity Index* and the *Cardiovascular Mortality Risk Score* and show a 3.4- and 2.2-times increased risk of cardiovascular and all-cause mortality if HRVI was ≤ 18.246, respectively. CI, confidence interval; HR, hazard ratio; HRVI, heart rate variability triangular index.

Kaplan-Meier plots of HRVI quartiles suggested a non-linear association between HRVI, cardiovascular, and all-cause mortality (Figures 2A,B). Figures 2C,D depict the hazard ratios relative to the median HRVI resulting from univariate non-linear Cox regression analysis for cardiovascular and all-cause mortality, respectively. Non-linear Cox regression analysis revealed a non-linear behavior of HRVI in univariate and adjusted analysis for cardiovascular mortality (Table 3) and all-cause mortality (Table 4). When categorizing HRVI into the inner and outer quartiles a value within the outer quartiles was associated with a 6.1- and 2.2-times increased risk for cardiovascular mortality and all-cause mortality in adjusted analysis, respectively. The 3-year cardiovascular mortality rate was 6.8 and 29.5% for the inner and outer HRVI quartile group, respectively, whilst the corresponding 6-year cardiovascular mortality rates were 11.4 and 36.4%, respectively (Figure 3A). For all-cause mortality the 3-year mortality rate was 38.6 and 61.4%, and the 6-year mortality rate was 65.9 and 81.8% for the inner and outer HRVI quartile group, respectively (Figure 3B). Figure 4 shows similar results when the cohort is stratified at the border of the first HRVI quartile. Exploratory analysis of HRVI numerator and denominator revealed heterogenous results (Supplementary Figure 1 and Supplementary Table 4).

**Table 3:** Linear and non-linear associations of risk variables with cardiovascular mortality in unadjusted and adjusted Cox regression analysis.

		**Linear term**				**Non-linear term**			
		**Unadjusted**		**Adjusted**		**Unadjusted**		**Adjusted**	
**Variable**	**Unit**	**HR (95% CI)**	***P***	**HR (95% CI)**	***P***	**HR (95% CI)**	***P***	**HR (95% CI)**	***P***
HRVI	1	–	–	–	–	NA	0.031	NA	0.032
HRVI (quartiles)	2 + 3 vs. 1 + 4	4.6 (1.7–12.6)	0.003	6.1 (2.1–17.7)	0.001	–	–	–	–
Cardiovascular mortality risk score	1 point	1.1 (0.9–1.2)	0.09	1.2 (1.0–1.3)	0.032	–	–	1.1 (0.9–1.2)	0.094
Charlson Comorbidity Index	1 point	1.1 (0.9–1.2)	0.29	1.1 (0.9–1.2)	0.54	–	–	1.0 (0.9–1.2)	0.64

**Table 4:** Linear and non-linear associations of risk variables with all-cause mortality in unadjusted and adjusted Cox regression analysis.

		**Linear term**				**Non-linear term**			
		**Unadjusted**		**Adjusted**		**Unadjusted**		**Adjusted**	
**Variable**	**Unit**	**HR (95% CI)**	***P***	**HR (95% CI)**	***P***	**HR (95% CI)**	***P***	**HR (95% CI)**	***P***
HRVI	1	–	–	–	–	NA	0.004	NA	0.003
HRVI (quartiles)	2 + 3 vs. 1 + 4	1.8 (1.1–3.0)	0.018	2.2 (1.3–3.8)	0.002	–	–	–	–
Cardiovascular mortality risk score	1 point	1.1 (1.0–1.2)	0.0004	1.1 (1.1–1.2)	0.0006	–	–	1.1 (1.0–1.2)	0.0008
Charlson Comorbidity Index	1 point	1.1 (1.0–1.2)	0.02	1.1 (0.9–1.2)	0.11	–	–	1.1 (0.09–1.2)	0.21

*Adjusted model for Charlson Comorbidity Index and Cardiovascular Mortality Risk Score. CI, confidence interval; NA, not applicable due to non-linear fitting of the models on two degrees of freedom on HRVI*.

C-index comparison of adjusted non-linear Cox regressions showed that the concordance of the Cox regression with the 24-h HRVI measurement was 7 and 5% higher than the mean concordances of 1000 Cox regressions with randomly selected 5 min HRVI segments for cardiovascular and all-cause mortality, respectively (cardiovascular mortality: 0.73 vs. 0.68 ± 0.02; all-cause mortality: 0.68 vs. 0.65 ± 0.01). Similarly, C-index comparison for 24-h SDNN measurements vs. 5 min segments differed by 12% for cardiovascular mortality (0.73 vs. 0.65 ± 0.01). Furthermore, when stratifying patients into sinus rhythm and AF, patients in sinus rhythm were descriptively more likely to have a significant *p*-value in the linear Cox regression model than AF patients (Supplementary Figure 2).

**Figure 5:**
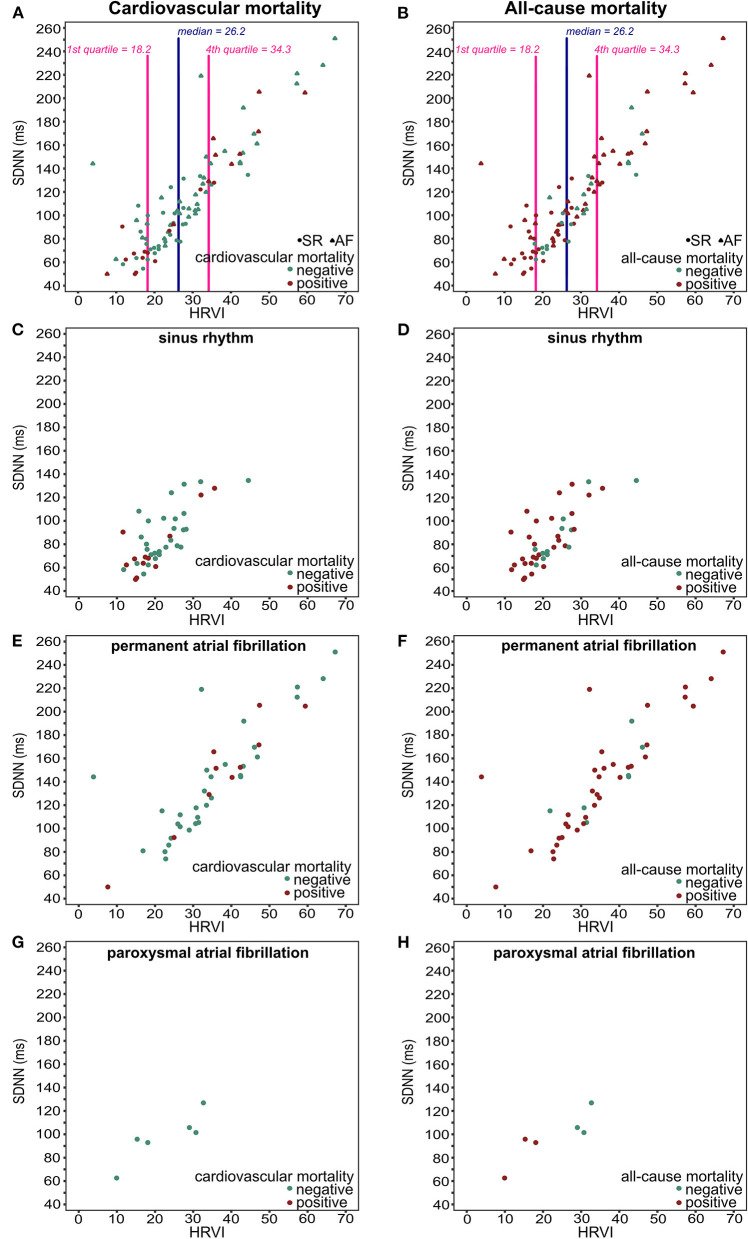
Scatter plots of HRVI and SDNN for all patients **(A,B)**, patients in sinus rhythm **(C,D)**, patients in permanent atrial fibrillation **(E,F)**, and patients in paroxysmal atrial fibrillation **(G,H)** displayed for cardiovascular mortality (left column), and for all-cause mortality (right column). HRVI, heart rate variability triangular index; SDNN, standard deviation of all NN intervals.

**Figure 6:**
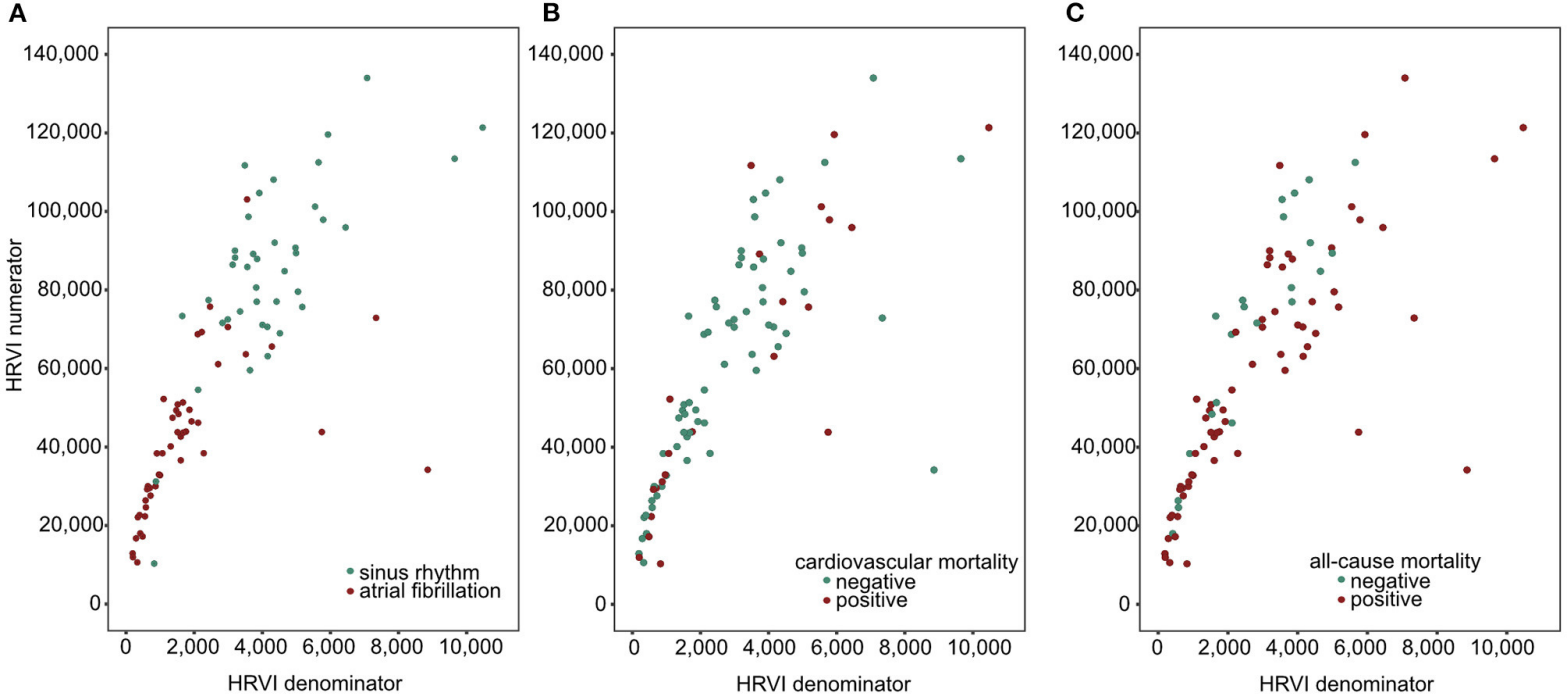
Scatter plots of HRVI numerator and denominator coded for atrial fibrillation or sinus rhythm **(A)**, cardiovascular **(B)**, and all-cause mortality **(C)**. HRVI, heart rate variability triangular index.

The median percentage of AF burden during 24-h recordings in the 48 patients with AF was 22.7% (6.6–45.5), 99.9% (85.8–99.9), 98.8% (88.0–99.9), and 99.8% (88.0–99.9) in the quartiles of HRVI (*p* = 0.008). *Post-hoc* analysis revealed significant difference of percentages between the first and the second (adjusted *p* = 0.03) and the first and the fourth quartile (adjusted *p* = 0.003). Figure 5 displays stratifications of patients in AF or sinus rhythm. Median follow up was 2.0 and 3.9 years for patients with AF or sinus rhythm, respectively. The mean heart rate was significantly higher in AF patients compared to patients in sinus rhythm (76.6 ± 14.2 vs. 68.7 ± 11.2 bpm, *p* = 0.006). However, patients with AF had significantly lower ratios of normal to all beats (39.8% [31.3–52.0] vs. 93.4% [85.3–96.5], *p* < 0.001). The median ratio of normal beats was 84.5% (71.3–95.6), 90.1% (52.7–96.4), 54.3% (45.1–92.4), and 32.7% (25.8–40.0) in the quartiles of HRVI (*p* < 0.001). *Post-hoc* analysis revealed significant differences of the fourth to the first, second or third quartile (each adjusted *p* < 0.001). Addition of the presence of AF or sinus rhythm or the normal beat ratio to the adjusted Cox regression models did not change the results (Tables 5, 6). Furthermore, only the HRVI numerator was associated with the presence of AF (odds ratio per 1,000 normal-to-normal intervals: 0.92, 95% confidence interval: 0.88–0.96, *p* < 0.001). Figure 6 displays the HRVI numerator and denominator with stratification for AF or sinus rhythm, cardiovascular and all-cause mortality.

**Table 5:** Sensitivity analysis of adjusted linear and non-linear Cox regression models additionally including the presence of atrial fibrillation (AF) or sinus rhythm (SR) for **(A)** cardiovascular and **(B)** all-cause mortality.

**Variable**	**Unit**	**Adjusted model**			
		**linear**		**Non-linear**	
		**HR (95% CI)**	***P***	**HR (95% CI)**	***P***
**(A) Cardiovascular mortality**					
HRVI	1	–	–	NA	0.027
HRVI (quartiles)	2 + 3 vs. 1 + 4	6.7 (2.2–19.9)	0.0007	–	–
Cardiovascular mortality risk score	1 point	1.2 (1.0–1.3)	0.020	1.1 (0.9–1.2)	0.08
Charlson Comorbidity Index	1 point	1.0 (0.9–1.2)	0.61	1.0 (0.9–1.2)	0.70
AF or SR	AF present	0.6 (0.3–1.5)	0.31	0.9 (0.3–2.3)	0.75
**(B) All-cause mortality**					
HRVI	1	–	–	NA	0.0034
HRVI (quartiles)	2 + 3 vs. 1+4	2.1 (1.3–3.6)	0.005	–	–
Cardiovascular mortality risk score	1 point	1.1 (1.0–1.2)	0.002	1.1 (1.0–1.2)	0.007
Charlson Comorbidity Index	1 point	1.1 (1.0–1.2)	0.09	1.1 (1.0–1.2)	0.09
AF or SR	AF present	1.3 (0.8–2.1)	0.36	1.8 (1.0–3.2)	0.06

*Adjusted model includes HRVI, the Charlson Comorbidity Index and Cardiovascular Mortality Risk Score, and atrial fibrillation vs. sinus rhythm. CI, confidence interval; HR, hazard ratio; HRVI, heart rate variability triangular index; NA, not applicable due to non-linear fitting of the models on two degrees of freedom on HRVI*.

**Table 6:** Sensitivity analysis of adjusted linear and non-linear Cox regression models additionally including the ratio of normal beats for **(A)** cardiovascular and **(B)** all-cause mortality.

**Variable**	**Unit**	**Adjusted model**			
		**linear**		**Non-linear**	
		**HR (95% CI)**	***P***	**HR (95% CI)**	***P***
**(A) Cardiovascular mortality**					
HRVI	1	–	–	NA	0.037
HRVI (quartiles)	2 + 3 vs. 1 + 4	6.4 (2.1–19.3)	0.001	–	–
Cardiovascular mortality risk score	1 point	1.2 (1.0–1.3)	0.030	1.1 (1.0–1.2)	0.15
Charlson Comorbidity Index	1 point	1.1 (0.9–1.2)	0.52	1.0 (0.9–1.2)	0.65
Ratio of normal beats	1%	1.4 (0.3–6.3)	0.70	0.4 (0.1–2.5)	0.35
**(B) All-cause mortality**					
HRVI	1	–	–	NA	0.003
HRVI (quartiles)	2 + 3 vs. 1 + 4	2.1 (1.2–3.7)	0.007	–	–
Cardiovascular mortality risk score	1 point	1.1 (1.0–1.2)	0.002	1.1 (1.0–1.2)	0.006
Charlson Comorbidity Index	1 point	1.1 (1.0–1.2)	0.12	1.1 (1.0–1.2)	0.21
Ratio of normal beats	1%	0.8 (0.3–2.0)	0.64	0.4 (0.1–1.0)	0.06

*Adjusted model includes HRVI, the Charlson Comorbidity Index and Cardiovascular Mortality Risk Score, and ratio of normal to overall beats. CI, confidence interval; HR, hazard ratio; HRVI, heart rate variability triangular index; NA, not applicable due to non-linear fitting of the models on two degrees of freedom on HRVI*.

Exploratory analysis of other HRV parameters that express overall long-term (SDNN, SDANN, ULF) and short-term (RMSSD) variability towards mortality revealed inconsistent results (Table 7).

**Table 7:** Linear and non-linear associations of risk variables with **(A)** cardiovascular mortality and **(B)** all-cause mortality in unadjusted and adjusted Cox regression analysis.

		**Linear term**				**Non-linear term**			
		**Unadjusted**		**Adjusted**		**Unadjusted**		**Adjusted**	
**Variable**	**Unit**	**HR (95% CI)**	***P***	**HR (95% CI)**	***P***	**HR (95% CI)**	***P***	**HR (95% CI)**	***P***
**(A) Cardiovascular mortality**									
SDNN	ms	0.9 (0.9–1.0)	0.92	0.9 (0.9–1.0)	0.62	NA	0.033	NA	0.032
SDANN	ms	0.9 (0.9–1.0)	0.36	1.0 (0.9–1.0)	0.25	NA	0.16	NA	0.21
RMSSD	ms	1.0 (0.9–1.0)	0.59	1.0 (0.9–1.0)	0.76	NA	0.26	NA	0.19
ULF	ms^2^	0.9 (0.9–1.0)	0.18	1.0 (1.0–1.0)	0.19	NA	0.072	NA	0.12
**(B) All-cause mortality**									
SDNN	ms	1.0 (0.9–1.0)	0.32	1.0 (1.0–1.0)	0.93	NA	0.17	NA	0.18
SDANN	ms	1.0 (1.0–1.0)	0.73	1.0 (1.0–1.0)	0.35	NA	0.031	NA	0.07
RMSSD	ms	1.1 (1.0–1.0)	0.049	1.0 (1.0–1.0)	0.14	NA	0.43	NA	0.35
ULF	ms^2^	1.0 (1.0–1.0)	0.18	1.0 (1.0–1.0)	0.15	NA	0.007	NA	0.037

*Adjusted model includes the predictor, the Charlson Comorbidity Index, and Cardiovascular Mortality Risk Score. CI, confidence interval; HR, hazard ratio; NA, not applicable; SDNN, standard deviation of all NN intervals; SDANN, standard deviation of the averages of NN intervals in all 5-min segments of 24 h recording; RMSSD, square root of the mean square of differences between adjacent NN intervals; ULF, ultra-low frequency*.

## Discussion

In this study, we found a U-shaped association of HRVI and mortality in hemodialysis AF patients. Stratification into inner and outer quartiles of HRVI was significantly associated with cardiovascular and all-cause mortality with a hazard ratio of 6.1 and 2.2 in adjusted models.

The left part of the U-shaped curve corresponds to depressed variability with impaired function of the autonomic nervous system which was primarily present in patients in sinus rhythm who had an AF history. According to the HRV Task Force, HRVI <15 is severely depressed (24). In the Swiss-AF study, dichotomizing the cohort at HRVI median of 14.3 led to association with mortality. In the present study, the cut-off of the first quartile was 18.2. When used to stratify patients below or above 18.2, patients below this cut-off were similarly exposed to increased mortality as when stratified by inner and outer HRVI quartiles. Furthermore, in our dataset it seems that HRVI values below the cohort median of 26.3, which comprises primarily patients in sinus rhythm, have an almost linear relationship to mortality. When randomly selecting 5 min segments, patients in sinus rhythm were more likely to have a significant *p*-value in the linear Cox regression model. HRVI has not yet been examined in hemodialyzed AF patients. In 120 hemodialysis patients without AF, HRVI was associated to cardiac death with a cohort median of 23.5 (26). Therefore, HRVI values might be higher in hemodialysis patients compared to patients without kidney disease.

The right part of the U-shaped curve could be explained by a higher prevalence of AF and a lower ratio of normal beats in the fourth HRVI quartile which is in line with a higher odds ratio for AF in case of lower HRVI numerator. Higher HRVI values correspond to a more diffuse pattern of RR intervals. Importantly, adding the presence of AF or the ratio of normal beats to the adjusted models did not change the non-linear association between HRVI and mortality. Linear relationships had been observed for patients without kidney disease with and without AF (17, 27). Limited evidence for a U-shaped behavior of HRV could be found and might be physiologically plausible (28). Dividing the HRVI in its numerator and denominator was not able to further explain the U-shaped profile. However, we assume that the U-shape displays rather irregular heart periods than an actual autonomic influence. Still, it is unclear if autonomic nervous system modulations influence the RR-interval irregularity in AF. Further studies have to confirm the non-linear risk relationships in AF patients on hemodialysis and to investigate whether disease-specific cut-offs exist. Furthermore, it might be of interest to examine HRVI in larger cohorts of only permanent AF patients. In general, however, examination of non-linear relationships should be considered when examining HRVI.

Patients in the Swiss-AF study were younger (73 vs. 76 years) and kidney disease was not present (17). Also, the Swiss-AF study recorded short-term ECGs of ≥5 min whereas we recorded 24-h ECGs. We found shorter recording periods to be less accurate for risk prediction than 24-h recordings. Most probably due to longer recording times in the present study, sinus rhythm in the ECG was present in 46% of our patients whereas in 58% of patients in the Swiss-AF study which might furthermore explain why we found a U-shaped association instead of a linear association between HRVI and mortality.

Compared to other HRV parameters that express overall variability only HRVI was able to predict both cardiovascular and all-cause mortality. Interestingly, other parameters such as SDNN and ULF also displayed a non-linear relationship to mortality. HRVI might be more robust and reproducible as it is, by design, less affected by artifacts and noise (29–31) and as it does not require artifact-clear ECG recognition (32).

One third of the dialysis cohort had either a medical history of AF or AF on the recorded ECG. This prevalence of AF in our cohort is comparable to similar hemodialysis populations (6). In 2% of patients, we made a new AF diagnosis. Although this might seem to represent a low proportion, higher morbidity and mortality in AF (6, 7) suggest that regular long-term ECG recordings should be considered since AF might be undetected in non-permanent recordings, e.g., by implantable loop recorders (2).

Hemodialysis patients with AF are characterized by a high morbidity and frailty (6, 7). Also, patients from our cohort were much sicker and older compared to hemodialysis patients without AF participating in the ISAR study. When dividing the cohort into inner and outer quartiles of HRVI, patients did not differ in comorbidities, age, and other factors. Interestingly, the *Charlson Comorbidity Index*, considered to have a strong predictive power of mortality in hemodialysis patients, was not associated with mortality in our data. In clinical practice, a detailed assessment of comorbidities generally allows approximate clinical estimates of individual risk. Nevertheless, this is unlikely sufficient in hemodialysis AF patients. Thus, HRVI has added benefit in hemodialysis AF patients with predicted risk independent of the *Cardiovascular Mortality Risk Score* (21).

Analysis of HRVI might also be possible in patients with a considerable AF burden in the recorded ECG. Furthermore, it seems particularly useful in AF patients. Consistent with other reports (33), we have not found an association of HRVI and cardiovascular mortality in hemodialysis patients without AF (34), even when considering non-linear associations (data not shown).

Finally, given the high risk of mortality in AF hemodialysis patients, establishing a clinical tool for identification of high-risk among this group is important. Results from this and previous trials might allow a general inclusion of AF patients into analyses of the autonomic nervous system when focusing on HRV parameters linked to the total variability (17, 18). Future studies should also investigate morbidities and instances of non-fatal hemorrhagic and thromboembolic strokes or bleeding events. Possibly, HRVI might also help in stratifying patients for oral anticoagulation which, in hemodialysis patients, is subject to an ongoing debate.

## Limitations

Limitations of the present study have to be considered. This is a rather small cohort, though one of the largest prospective dialysis cohorts where 24-h ECGs are available. The method of HRV calculation which was originally only developed for analyses in sinus rhythm ECGs was also used in AF patients. Therefore, it has to be taken into account that lower percentages of normal beats in AF and arrhythmic patterns that can make it difficult to discriminate normal heart beats might limit the interpretation of HRVI in AF patients. Sex differences are well-known in AF (35). In our cohort, sex distribution was not balanced between the groups and some sex differences were present. However, the sex distribution of our cohort is typical for a hemodialysis cohort. Furthermore, adding sex to the Cox regression models did not change the results (data not shown). The high frailty in our cohort limited the number of available ECGs. Due to a low number of cardiovascular events, adjusted Cox regression analysis was limited.

## Conclusion

In conclusion, the study found a non-linear association of HRVI to cardiovascular and all-cause mortality independent of known strong risk factors in hemodialysis AF patients. Stratification into inner and outer quartiles of HRVI identified high-risk hemodialysis AF patients, who are known to be at increased mortality risk compared to hemodialysis patients without AF.

## Data Availability Statement

The datasets for this manuscript are not publicly available because written informed consent did not include wording on data sharing (German data protection laws). Reasonable requests to access the datasets should be directed to Matthias C. Braunisch, Matthias.Braunisch@mri.tum.de.

## Ethics Statement

The studies involving human participants were reviewed and approved by Medical Ethics Committee of the Klinikum Rechts der Isar of the Technical University Munich and Bavarian State Board of Physicians. The patients/participants provided their written informed consent to participate in this study.

## Author Contributions

This article was conceptualized by MB and CS. MB wrote the first draft of the manuscript. MB, SWe, and BH performed the statistical analysis. MB, GL, RG, JM, QB, ST, MS, ML, CH-L, TA, and MP contributed to the data acquisition. Raw ECG data processing was performed by CM, SWa, and AM. CS, AB, CK, LR, GS, UH, and MM contributed to the supervision. All co-authors were involved with data interpretation, revising the work, and provided important intellectual content. All authors have seen and approved the final manuscript.

## Funding

This work was supported by the Klinikum Rechts der Isar of the Technical University of Munich.

## Conflict of Interest

MB reports received personal fees from Vifor Pharma unrelated to the project. MP is employed by Nephrocare. CM and SWa are employed by the Austrian Institute of Technology GmbH, a non-profit research organization. The remaining authors declare that the research was conducted in the absence of any commercial or financial relationships that could be construed as a potential conflict of interest.

## Publisher's Note

All claims expressed in this article are solely those of the authors and do not necessarily represent those of their affiliated organizations, or those of the publisher, the editors and the reviewers. Any product that may be evaluated in this article, or claim that may be made by its manufacturer, is not guaranteed or endorsed by the publisher.
