# Supplementary material for: U-Shaped Association of the Heart Rate Variability Triangular Index and Mortality in Hemodialysis Patients With Atrial Fibrillation
Source: Front Cardiovasc Med. 2021 Nov 29;8:751052. doi: 10.3389/fcvm.2021.751052 (PMC8667023; doi:10.3389/fcvm.2021.751052)
Supplement: Supplementary file 1 [file Data_Sheet_1.docx]

**U-shaped Association of the Heart Rate Variability Triangular Index and Mortality in Hemodialysis Patients with Atrial Fibrillation**

**Supplement**

Matthias C. Braunisch^1,#^, Christopher C. Mayer^2^, Stanislas Werfel^1^, Axel Bauer^3,4^, Bernhard Haller^5^, Georg Lorenz^1^, Roman Günthner^1^, Julia Matschkal^1^, Quirin Bachmann^1^, Stephan Thunich^1,6^, Michaela Schlegl^1^, Maximilian Ludwig^1^, Christopher Holzmann-Littig^1^, Tarek Assali^1^, Martin Pachmann^7^, Claudius Küchle^1^, Lutz Renders^1^, Siegfried Wassertheurer^2^, Alexander Müller^8^, Georg Schmidt^8^, Uwe Heemann^1^, Marek Malik^9,10^, and Christoph Schmaderer^1,#^

^1^ Technical University of Munich, Germany; School of Medicine, Department of Nephrology, Klinikum rechts der Isar

^2^ Center for Health & Bioresources, Biomedical Systems, AIT Austrian Institute of Technology GmbH, Vienna, Austria

^3^ University Hospital for Internal Medicine III, Medical University Innsbruck, Innsbruck, Austria

^4^ Department of Cardiology, Munich University Clinic, DZHK (German Centre for Cardiovascular Research), Ludwig-Maximilians University, Munich, Germany

^5^ Technical University of Munich, Germany; School of Medicine, Institute of Medical Informatics, Statistics and Epidemiology (IMedIS), Klinikum rechts der Isar

^6^ Technical University of Munich, Germany; School of Medicine, Deutsches Herzzentrum München

^7^ Nephrocare DIZ-München, Rindermarkt, Munich, Germany

^8^ Technical University of Munich, Germany; School of Medicine, Klinik für Innere Medizin I, Klinikum rechts der Isar

^9^ National Heart and Lung Institute, Imperial College London, London, United Kingdom

^10^ Department of Internal Medicine and Cardiology, Faculty of Medicine, Masaryk University, Brno, Czech Republic

# Supplemental Tables and Figures

| **Supplementary Table 1.** Baseline characteristics stratified by sex. | | | |  |  |
| --- | --- | --- | --- | --- | --- |
|  |  | **Sex** | |  |  |
|  |  | **Male (n=63)** | **Female (n=25)** | | ***P*** |
| Age (years) |  | 75.3 (±9.1) | 76.8 (±6.9) |  | 0.44 |
| Body mass index (kg/m2) | | 25.7 (23.4 - 28.0) | 24.7 (22.9 - 29.8) | | 0.83 |
| Dialysis vintage (months) | | 24.0 (12.0 - 43.5) | 50.0 (28.0 - 82.0) | | 0.007 |
| Ultrafiltration rate (mL/h) | | 524.6 (±296.2) | 503.8 (±209.2) | | 0.75 |
| Net ultrafiltration (L) | | 1.8 (±1.4) | 1.5 (±1.0) |  | 0.40 |
| Heart rate (bpm) | | 73.9 (±13.6) | 70.8 (±13.2) |  | 0.33 |
| Systolic blood pressure (mmHg) | | 127.0 (±22.0) | 139.2 (±31.6) | | 0.043 |
| Diastolic blood pressure (mmHg) | | 68.3 (±15.2) | 67.0 (±15.7) |  | 0.74 |
| Kt/V |  | 1.30 (±0.29) | 1.45 (±0.37) |  | 0.053 |
| Blood urea nitrogen (mg/dL) | | 58.7 (±17.6) | 58.2 (±17.1) |  | 0.90 |
| Phosphate (mmol/L) | | 1.60 (1.35 - 1.95) | 1.80 (1.65 - 2.06) | | 0.066 |
| Total calcium (mmol/L) | | 2.29 (±0.17) | 2.35 (±0.15) |  | 0.14 |
| Calcium *x* phosphate (mmol^2^/L^2^) | | 3.95 (±1.86) | 4.33 (±1.04) |  | 0.34 |
| Creatinine (mg/dL) | | 7.2 (±2.6) | 6.9 (±1.5) |  | 0.63 |
| High-sensitivity CRP (mg/dL) | | 0.62 (0.27 - 1.32) | 0.42 (0.19 - 0.65) | | 0.086 |
| Albumin (g/dL) | | 3.90 (3.63 - 4.19) | 3.90 (3.82 - 4.18) | | 0.30 |
| Parathyroid hormone (pg/mL) | | 257.1 (130.0 - 360.5) | 195.0 (127.0 - 316.8) | | 0.46 |
| Leukocytes (G/L) | | 6.92 (±2.17) | 7.22 (±1.69) |  | 0.54 |
| Total cholesterol (mg/dL) | | 174.8 (±52.7) | 171.0 (±34.2) | | 0.75 |
| Charlson Comorbidity Index (0 to 21) | | 6.0 (4.0 - 8.0) | 5.0 (3.0 - 7.0) | | 0.13 |
| Cardiovascular mortality risk score (-11 to 39) | | 14.0 (10.5 - 17.0) | 13.0 (9.0 - 16.0) | | 0.10 |
| Diabetes mellitus | | 28 (44.4%) | 10 (40.0%) |  | 0.81 |
| History of myocardial infarction | | 22 (34.9%) | 3 (12.0%) |  | 0.037 |
| Left ventricular hypertrophy | | 21 (33.3%) | 9 (36.0%) |  | 0.81 |
| Left ventricular ejection fraction (%), n=22 | | 47 (±17) | 49 (±11) |  | 0.78 |
| Heart failure |  | 20 (31.7%) | 5 (20.0%) |  | 0.31 |
| Peripheral artery disease | | 21 (33.3%) | 5 (25.0%) |  | 0.30 |
| Hypertension | | 60 (95.2%) | 24 (96.0%) |  | 1.00 |
| Coronary heart disease | | 34 (54.0%) | 5 (20.0%) |  | 0.004 |
| Cerebrovascular disease | | 11 (17.4%) | 6 (24.0%) |  | 0.55 |
| Smoking (ever) | | 14 (22.2%) | 0 (0.0%) |  | 0.008 |
| CHA2DS2-VASc score | | 4.0 (3.0 - 5.0) | 4.0 (3.0 - 5.0) | | 0.63 |
| Results are presented as mean (±SD) and median (interquartile range) for normally and non-normally distributed data, respectively; categorical data as total number (percentage). *P*-values present the results of group-wise comparisons of sexes. CHA_2_DS_2_-VASc, Congestive heart failure, Hypertension, Age ≥75 years, Diabetes mellitus, Stroke, Vascular disease, Age 65-74 years, Sex category (female). | | | | | |

| **Supplementary Table 2.** Comparison of study population to excluded patients. | | | | |
| --- | --- | --- | --- | --- |
|  | **Study population** | |  |  |
|  | **Excluded (n=431)** | **Included (n=88)** |  | ***P*** |
| Age (years) | 65.2 (51.8 - 75.2) | 76.4 (70.6 - 80.6) |  | < 0.001 |
| Sex (female) | 136 (31.6%) | 25 (28.4%) |  | 0.61 |
| Body mass index (kg/m^2^) | 25.0 (22.6 - 28.7) | 25.6 (23.0 - 28.4) |  | 0.50 |
| Dialysis vintage (months) | 47.0 (25.0 - 81.5) | 27.0 (13.8 - 60.2) |  | < 0.001 |
| Ultrafiltration rate (mL/h) | 500.0 (325.8 - 674.9) | 509.2 (369.3 - 691.4) |  | 0.40 |
| Net ultrafiltration (L) | 1.8 (±1.2) | 1.7 (±1.3) |  | 0.62 |
| Heart rate (bpm) | 73.5 (65.8 - 80.6) | 71.7 (62.7 - 81.0) |  | 0.20 |
| Systolic blood pressure (mmHg) | 135.0 (121.0 - 149.0) | 129.0 (116.8 - 146.5) |  | 0.055 |
| Diastolic blood pressure (mmHg) | 73.3 (±14.6) | 67.9 (±15.3) |  | 0.002 |
| Kt/V | 1.47 (1.25 - 1.69) | 1.35 (1.15 - 1.56) |  | < 0.001 |
| Blood urea nitrogen (mg/dL) | 61.7 (±16.5) | 58.5 (±17.4) |  | 0.10 |
| Phosphate (mmol/L) | 1.68 (1.39 - 2.04) | 1.68 (1.39 - 2.01) |  | 0.70 |
| Total calcium (mmol/L) | 2.26 (2.15 - 2.38) | 2.31 (2.21 - 2.42) |  | 0.019 |
| Calcium *x* phosphate (mmol^2^/L^2^) | 3.78 (3.12 - 4.64) | 3.77 (3.06 - 4.58) |  | 0.92 |
| Creatinine (mg/dL) | 8.7 (±2.8) | 7.1 (±2.3) |  | < 0.001 |
| High-sensitivity CRP (mg/dL) | 0.41 (0.18 - 0.94) | 0.58 (0.22 - 1.20) |  | 0.12 |
| Albumin (g/dL) | 4.00 (3.80 - 4.20) | 3.90 (3.70 - 4.20) |  | 0.073 |
| Parathyroid hormone (pg/mL) | 219.8 (104.0 - 394.5) | 226.5 (128.5 - 359.6) |  | 0.97 |
| Leukocytes (G/L) | 6.70 (5.45 - 8.00) | 6.95 (5.50 - 8.40) |  | 0.56 |
| Total cholesterol (mg/dL) | 176.0 (146.0 - 204.0) | 169.0 (139.0 - 200.0) |  | 0.25 |
| Charlson Comorbidity Index (0 to 21) | 3.0 (1.0 - 5.0) | 6.0 (4.0 - 8.0) |  | < 0.001 |
| Cardiovascular mortality risk score (-11 to 39) | 10.0 (5.0 - 14.0) | 14.0 (10.0 - 17.0) |  | < 0.001 |
| Diabetes mellitus | 171 (39.7%) | 38 (36.4%) |  | 0.55 |
| History of myocardial infarction | 78 (18.1%) | 25 (28.4%) |  | 0.039 |
| Left ventricular hypertrophy | 122 (28.3%) | 30 (34.1%) |  | 0.30 |
| Heart failure | 74 (17.2%) | 25 (28.4%) |  | 0.017 |
| Peripheral artery disease | 97 (22.5%) | 26 (29.5%) |  | 0.17 |
| Hypertension | 405 (94.0%) | 84 (95.5%) |  | 0.80 |
| Coronary heart disease | 148 (34.3%) | 39 (44.3%) |  | 0.088 |
| Cerebrovascular disease | 68 (15.8%) | 17 (19.3%) |  | 0.43 |
| Smoking (ever) | 104 (24.1%) | 14 (15.9%) |  | 0.062 |
| Results are presented as mean (±SD) and median (IQR) for normally and non-normally distributed data, respectively; categorical data as total number (percentage). *P*-values present the results of group-wise comparisons of patients that were excluded or included. | | | | |

| **Supplementary Table 3.** Specific causes of cardiovascular and all-cause mortality. | |
| --- | --- |
| **Cardiovascular events (n=22)** | n |
| Sudden cardiac death | 7 |
| Myocardial infarction | 2 |
| Heart failure | 5 |
| Major stroke | 2 |
| Cardiac surgical procedure | 2 |
| Pulmonary embolism | 1 |
| Aortal dissection | 1 |
| Peripheral ischemic event | 2 |
| **Non-cardiovascular events (n=44)** | |
| Infectious events | 21 |
| Malignant disease | 6 |
| Withdrawal from treatment | 4 |
| Suicide | 2 |
| Respiratory failure | 1 |
| Volvulus of the small intestine | 1 |
| Multiorgan failure | 1 |
| Unknown | 8 |
|  |  |

| **Supplementary Table 4**. Exploratory analysis of linear and nonlinear Cox regression for univariate and adjusted models with HRVI numerator and denominator for **A** cardiovascular and **B** all-cause mortality. | | | | | | | | | | | | |
| --- | --- | --- | --- | --- | --- | --- | --- | --- | --- | --- | --- | --- |
|  |  | **Linear term** | | | | |  | **Nonlinear term** | | | | |
|  |  | **HRVI numerator** | |  | **HRVI denominator** | |  | **HRVI numerator** | |  | **HRVI denominator** | |
| **Model** | **Unit** | **HR (95% CI)** | ***P*** |  | **HR (95% CI)** | ***P*** |  | **HR (95% CI)** | ***P*** |  | **HR (95% CI)** | ***P*** |
| **A. Cardiovascular mortality** | | |  |  |  |  |  |  |  |  |  |  |
| Univariate | 1,000 | 0.99 (0.98-1.01) | 0.32 |  | 1.07 (0.87-1.32) | 0.51 |  | NA | 0.072 |  | NA | 0.039 |
| Adjusted | 1,000 | 0.99 (0.98-1.01) | 0.53 |  | 1.12 (0.91-1.37) | 0.29 |  | NA | 0.096 |  | NA | 0.058 |
| **B. All-cause mortality** | | |  |  |  |  |  |  |  |  |  |  |
| Univariate | 1,000 | 0.99 (0.98-0.99) | 0.019 |  | 1.01 (0.89-1.15) | 0.91 |  | NA | 0.11 |  | NA | 0.012 |
| Adjusted | 1,000 | 0.99 (0.98-1.00) | 0.10 |  | 1.06 (0.94-1.21) | 0.34 |  | NA | 0.22 |  | NA | 0.042 |
| Adjusted model includes HRVI numerator or denominator, the *Charlson Comorbidity Index* and *Cardiovascular Mortality Risk Score*, and atrial fibrillation vs sinus rhythm. Abbreviations: CI, confidence interval; HR, hazard ratio; HRVI, heart rate variability triangular index; NA, not applicable due to nonlinear fitting of the models on two degrees of freedom on HRVI. | | | | | | | | | | | | |

**Supplementary Figure 1.** Exploratory analysis of nonlinear associations of HRVI numerator and denominator and mortality. Univariate association of HRVI numerator and denominator for cardiovascular morality (A, C) and for all-cause mortality (B, C). Only the denominator was significantly associated with mortality, detailed results of the nonlinear Cox regressions can be found in Supplementary Table 4.

Abbreviations: HRVI, heart rate variability triangular index.

**Supplementary Figure 2**. Histograms of *p*-values of 1000 univariate linear Cox regressions with randomly selected 5 minutes HRVI intervals for all patients and stratified for patients in sinus rhythm (n=40) and atrial fibrillation (n=48) displayed for cardiovascular mortality (left column), and for all-cause mortality (right column).
